# Non-Malignant and Malignant Tumors

**Published:** 1888-04

**Authors:** J. N. Martin


### Non-Malignant and Malignant Tumors. Differential Points.

J. N. MARTIN, M.D.

Lecturer on Dental Pathology, Surgery and Therapeutics, in the Dental College of the University of Michigan.

If cancer, and other malignant tumors are amenable to treatment at all, it is in the early stage of the disease. Some of our best authorities believe that malignant growths in the earliest, or incipient stage, can be removed with reasonable hope of their not returning; they regard these growths as local at first in many cases, while in later stages the malignant cells, or viruses, are conveyed to other parts by the lymphatics and by a process of inoculation or grafting new foci are started; when this occurs the disease is no longer local, and then, even if the original mass is removed, one or more of the infected districts begins to develop; this may occur near the original seat or in some part quite distant, but the disease usually manifests itself in the nearest chain of lymphatic glands first; but a malignant growth after removal from the jaw, or other oral structures, may develop in some of the vital organs, as the liver, lungs, or stomach.

It is important for dentists to keep in mind the differential points between non-malignant and malignant growths about the mouth, as they are often consulted in regard to these growths and especially tumors about the maxillæ; and infrequently when not consulted with reference to these growths they have opportunities to observe them in the incipient stage.

One who is able to diagnose correctly in these cases adds much to himself, and confers a boon upon his patient, by either dispelling ungrounded fears, or advises early and radical treatment in case of malignancy.

In order to give concisely the differential points between non-malignant and malignant tumors I give the following tabulated form:

| NON-MALIGNANT. | MALIGNANT. |
| --- | --- |
| 1. Develop *slowly* and cease growing after a time and at certain size generally. | 1. When once started, they grow *rapidly*, with no tendency to stop growing. |
| 2. The surrounding structures are not involved; tumor generally clearly outlined. | 2. Surrounding structures are involved, and there is so much infiltration that it is difficult to tell where healthy normal structures begin. |
| 3. Generally do not ulcerate or break down, but may do so in later stages from an active cause. | 3. Great tendency to ulceration, and when started there is no tendency to stop. Tissues about changed from the normal. |
| 4. Ulceration stops when cause of irritation is removed and reparative process begins. | 4. Reparative process has no effect—degeneration continues. |
| 5. Constitution generally not affected. | 5. Constitution much affected, especially in later stages when health is undermined. |
| 6. Structure of tumor like other tissues in the body, as fibrous, nervous, and cartilaginous. | 6. It is a new formation and unlike other tissues in the body which are normal. |

The microscope must decide the differences in the last division.

